# Supplementary material for: Undergraduate data science degrees emphasize computer science and statistics but fall short in ethics training and domain-specific context
Source: PeerJ Comput Sci. 2021 Mar 25;7:e441. doi: 10.7717/peerj-cs.441 (PMC8022506; doi:10.7717/peerj-cs.441)
Supplement: Supplemental Information 1 [file peerj-cs-07-441-s001.docx]

**Supplemental Table 1. Areas and sub-areas of the NASEM framework.**

| **Area** | **Sub-area** |
| --- | --- |
| Mathematical foundations | Set theory and basic logic |
|  | Multivariate thinking via functions |
|  | Basic probability theory |
|  | Matrices and basic linear algebra |
|  | Networks and graph theory |
|  | Optimization |
| Computational foundations | Basic abstractions |
|  | Algorithmic thinking |
|  | Programming concepts |
|  | Data structures |
|  | Simulations |
| Statistical foundations | Variability, uncertainty, sampling error, and inference |
|  | Multivariate thinking |
|  | Nonsampling error, design, experiments (e.g., A/B testing), biases, confounding, and causal inference |
|  | Exploratory data analysis |
|  | Statistical modeling and model assessment |
|  | Simulations and experiments |
| Data management and curation | Data provenance |
|  | Data preparation, especially data cleansing and data transformation |
|  | Data management |
|  | Record retention policies |
|  | Data subject privacy |
|  | Missing and conflicting data |
|  | Modern databases |
| Data description and visualization | Data consistency checking |
|  | Exploratory data analysis |
|  | Grammar of graphics |
| Data modeling and assessment | Machine learning |
|  | Multivariate modeling and supervised learning |
|  | Dimension reduction techniques and unsupervised learning |
|  | Deep learning |
|  | Model assessment and sensitivity analysis |
|  | Model interpretation (particularly for black box models) |
| Workflow and reproducibility | Workflows and workflow systems |
|  | Reproducible analysis |
| Communication and teamwork | Ability to understand client needs |
|  | Clear and comprehensive reporting |
|  | Conflict resolution skills |
|  | Well-structured technical writing without jargon |
|  | Effective presentation skills |
| Domain-specific considerations |  |
| Ethical problem solving | Ethical precepts for data science and codes of conduct |
|  | Privacy and confidentiality |
|  | Responsible conduct of research |
|  | Ability to identify “junk” science |
|  | Ability to detect algorithmic bias |
