# Supplementary material for: Undergraduate data science degrees emphasize computer science and statistics but fall short in ethics training and domain-specific context
Source: PeerJ Comput Sci. 2021 Mar 25;7:e441. doi: 10.7717/peerj-cs.441 (PMC8022506; doi:10.7717/peerj-cs.441)
Supplement: Supplemental Information 2 [file peerj-cs-07-441-s002.docx]

**Supplemental Table 2. Areas and sub-areas of the GDS framework.**

| **Area** | **Sub-area** |
| --- | --- |
| Data gathering, preparation, and exploration |  |
| Data representation and transformation | Modern databases |
|  | Mathematical representations |
| Computing with data |  |
| Data modeling | Generative modeling |
|  | Predictive modeling |
| Data visualization and presentation |  |
| Science about data science |  |
