# Supplementary material for: Undergraduate data science degrees emphasize computer science and statistics but fall short in ethics training and domain-specific context
Source: PeerJ Comput Sci. 2021 Mar 25;7:e441. doi: 10.7717/peerj-cs.441 (PMC8022506; doi:10.7717/peerj-cs.441)
Supplement: Supplemental Information 3 — URLs for program information and date the URL was last checked. [file peerj-cs-07-441-s003.docx]

**Supplemental Table 3. Information sources for undergraduate data science programs considered in this work.** URLs for program information and date the URL was last checked.

| **Institution** | **Program** | **URL** | **Date** |
| --- | --- | --- | --- |
| Ohio State University - Main Campus | B.S. in Data Analytics | <https://data-analytics.osu.edu/major/core-curriculum> | 2019-10-28 |
| Pennsylvania State University - Main Campus | B.S. in Data Sciences, Applied Data Science Option | <https://ist.psu.edu/students/undergrad/majors/ds/ads> | 2019-11-07 |
| Pennsylvania State University - Main Campus | B.S. in Data Sciences, Computational Science Option | <https://www.eecs.psu.edu/students/undergraduate/Data-Sciences.aspx> | 2019-11-05 |
| Stanford University | Minor in Data Science | <https://statistics.stanford.edu/data-science-minor> | 2019-11-07 |
| University of Arizona | B.A. in Statistics and Data Science | <https://www.math.arizona.edu/academics/undergrads/requirements/SDS> | 2019-10-08 |
| University of Arizona | B.S. in Statistics and Data Science | <https://www.math.arizona.edu/academics/undergrads/requirements/SDS> | 2019-12-06 |
| University of California - Berkeley | B.A. in Data Science | <https://data.berkeley.edu/academics/undergraduate-programs/data-science-major> | 2019-11-07 |
| University of California - Davis | B.S. in Statistics, Statistical Data Science Track | <https://statistics.ucdavis.edu/undergrad/bs-statistical-data-science-track> | 2019-10-24 |
| University of Colorado - Boulder | B.A. in Statistics and Data Science | <https://www.colorado.edu/amath/academics/undergraduate-program/statistics-and-data-science-major-ba> | 2019-11-08 |
| University of Illinois at Urbana-Champaign | Certificate in Data Science | <https://stat.illinois.edu/academics/undergraduate-program/degree-programs/certificate-data-science> | 2019-11-04 |
| University of Iowa | B.S. in Data Science | <https://stat.uiowa.edu/undergraduate-programs/data-science/data-science> | 2019-10-28 |
| University of Maryland - College Park | B.S. in Computer Science, Data Science Specialization | <https://undergrad.cs.umd.edu/degree-requirements-cs-major#DataScience> | 2019-11-04 |
| University of Washington - Seattle | B.S. in Applied & Computational Mathematical Sciences, Data Sciences & Statistics Track | <https://acms.washington.edu/content/data-sciences-and-statistics> | 2019-11-20 |
| University of Washington - Seattle | B.S. in Computer Science, Data Science Option | <https://www.cs.washington.edu/academics/ugrad/courses/requirements> | 2019-11-20 |
| University of Washington - Seattle | B.S. in Human Centered Design & Engineering, Data Science Option | <https://www.hcde.washington.edu/bs/current/requirements> | 2019-11-21 |
| University of Washington - Seattle | B.S. in Informatics, Data Science Track | <https://ischool.uw.edu/programs/informatics/curriculum> | 2019-11-20 |
| University of Washington - Seattle | B.S. in Statistics, Data Science Option | <https://www.stat.washington.edu/academics/undergraduate/major/> | 2019-11-20 |
| Washington State University | B.S. in Data Analytics | <https://data-analytics.wsu.edu/197-2/core-courses/> | 2019-12-16 |
