# Supplementary material for: Undergraduate data science degrees emphasize computer science and statistics but fall short in ethics training and domain-specific context
Source: PeerJ Comput Sci. 2021 Mar 25;7:e441. doi: 10.7717/peerj-cs.441 (PMC8022506; doi:10.7717/peerj-cs.441)
Supplement: Supplemental Information 4 — Values are for Tukey-corrected pairwise comparisons between areas in ordinal regression model (above diagonal: p-values; below diagonal: z-scores). Shaded cells indicate comparisons with adjusted p < 0.05. [file peerj-cs-07-441-s004.docx]

**Supplemental Table 4. Post-hoc comparisons among areas in the NASEM framework.** Values are for Tukey-corrected pairwise comparisons between areas in ordinal regression model (above diagonal: p-values; below diagonal: z-scores). Shaded cells indicate comparisons with adjusted p < 0.05.

|  | Data description & visualization | Computational foundations | Statistical foundations | Data modeling & assessment | Mathematical foundations | Communication & teamwork | Data management & curation | Domain-specific considerations | Workflow & reproducibility | Ethical problem solving |
| --- | --- | --- | --- | --- | --- | --- | --- | --- | --- | --- |
| Data description & visualization |  | 0.9995 | 0.5304 | 0.2344 | 0.0048 | < 0.0001 | < 0.0001 | 0.0003 | < 0.0001 | < 0.0001 |
| Computational foundations | 0.706 |  | 0.0705 | 0.0139 | < 0.0001 | < 0.0001 | < 0.0001 | < 0.0001 | < 0.0001 | < 0.0001 |
| Statistical foundations | 2.096 | 3.047 |  | 0.9999 | 0.4519 | 0.0086 | < 0.0001 | 0.0204 | < 0.0001 | < 0.0001 |
| Data modeling & assessment | 2.565 | 3.555 | -0.565 |  | 0.8012 | 0.0486 | < 0.0001 | 0.0511 | < 0.0001 | < 0.0001 |
| Mathematical foundations | 3.841 | 4.875 | -2.207 | -1.691 |  | 0.947 | 0.0005 | 0.4523 | 0.0009 | < 0.0001 |
| Communication & teamwork | -5.030 | -6.183 | -3.687 | -3.173 | -1.330 |  | 0.0280 | 0.8777 | 0.0137 | < 0.0001 |
| Data management & curation | 7.315 | 8.526 | -6.597 | -6.179 | -4.380 | 3.349 |  | 1.0000 | 0.8917 | 0.5797 |
| Domain-specific considerations | 4.511 | 5.125 | -3.444 | -3.157 | -2.206 | 1.536 | 0.239 |  | 0.9569 | 0.9150 |
| Workflow & reproducibility | 6.575 | 7.321 | -5.654 | 5.354 | 4.259 | 3.559 | 1.502 | 1.286 |  | 1 |
| Ethical problem solving | 8.388 | 9.509 | -7.824 | -7.467 | -5.875 | 5.029 | -2.027 | 1.439 | 0.068 |  |
