# Supplementary material for: Undergraduate data science degrees emphasize computer science and statistics but fall short in ethics training and domain-specific context
Source: PeerJ Comput Sci. 2021 Mar 25;7:e441. doi: 10.7717/peerj-cs.441 (PMC8022506; doi:10.7717/peerj-cs.441)
Supplement: Supplemental Information 5 — Values are for Tukey-corrected pairwise comparisons between areas in ordinal regression model (above diagonal: p-values; below diagonal: z-scores). Shaded cells indicate comparisons with adjusted p < 0.05. [file peerj-cs-07-441-s005.docx]

**Supplemental Table 5. Post-hoc comparisons among areas in the GDS framework.** Values are for Tukey-corrected pairwise comparisons between areas in ordinal regression model (above diagonal: p-values; below diagonal: z-scores). Shaded cells indicate comparisons with adjusted p < 0.05.

|  | Computing with data | Data modeling | Data visualization & presentation | Data representation & transformation | Data gathering, preparation, & exploration | Science about data science |
| --- | --- | --- | --- | --- | --- | --- |
| Computing with data |  | 0.9770 | 0.8345 | 0.6630 | 0.0310 | 0.0001 |
| Data modeling | 0.7400 |  | 0.9863 | 0.9024 | 0.0186 | < 0.0001 |
| Data visualization & presentation | -1.2050 | -0.6590 |  | 0.9998 | 0.2278 | 0.0002 |
| Data representation & transformation | 1.5020 | 1.0460 | 0.2640 |  | 0.2009 | < 0.0001 |
| Data gathering, preparation, & exploration | 3.0130 | -3.1780 | 2.2210 | -2.2830 |  | 0.0687 |
| Science about data science | 4.6440 | 5.3830 | 4.2990 | 4.6590 | 2.7340 |  |
