# Supplementary material for: Undergraduate data science degrees emphasize computer science and statistics but fall short in ethics training and domain-specific context
Source: PeerJ Comput Sci. 2021 Mar 25;7:e441. doi: 10.7717/peerj-cs.441 (PMC8022506; doi:10.7717/peerj-cs.441)
Supplement: Supplemental Information 6 — Rubric used to evaluate undergraduate data science programs in NA framework and GDS framework. [file peerj-cs-07-441-s006.docx]

Appendix A: Coding Rubric

General Overview: Only course titles and descriptions are considered in assigning scores. The scoring scale breaks down as follows:

4: Student is well-versed in the area, with at least one required course covering the area;

3: Student has some knowledge of the area, although likely only at rudimentary level;

2: Student may have been exposed to this area through an optional course satisfying a

requirement;

1: No expectation that the student is familiar in this area.

Notes: Not all subareas have a fully-defined, four-point scoring. That is, for some subareas, only criteria for scores of 4, 2, and 1 are defined. Furthermore, for the following subareas of the NA framework, the lowest score possible is 2, based on the assumption that a student graduating with a bachelor’s degree would receive at least some training in these areas: "Effective presentation skills;" "Well-structured technical writing without jargon;" "Conflict resolution skills;" and "Clear and comprehensive reporting."

Regarding optional courses, given that the purpose of the coding rubric is to assess a student’s competency for a particular area, the number of options for a given requirement is taken into account. That is, if the number of optional courses satisfying the criterion for the area made up at least 10% of the listed courses, then a score of 2 is given; otherwise, a score of 1 is assigned. Additionally, for any list with over 20 optional courses, regardless of the percentage of courses the students would be required to take, a score of 1 is given. Finally, an optional course within a specialization for a degree receives a score of 1. For example, Ohio State University’s B.S. in Data Analytics has five possible specializations within the major; a score of 1 is assigned if an area within the frameworks is satisfied by an optional course within a specialization.

**GDS framework, after Donoho (2017)**

1. Data Gathering, Preparation, and Exploration

4: Required course(s) covering all of the following areas: data gathering, data collection, or using data from public sources; data preparation; and data exploration.

3: Required course(s) covering two of the following three areas: data gathering, data

collection, or using data from public sources; data preparation; and data exploration.

2: Required introductory statistics course, without additional explanation; or required or optional course covering at least one of the following three areas: data gathering, data collection, or using data from public sources; data preparation; and data exploration.

1: No course covering material.

2. Data Representation and Transformation

- Modern Databases

4: Required course description, if not the course title, includes the term "database" or named specific types of databases, including SQL (or MySQL, NoSQL, SQLite, etc.) or graph databases.

2: Optional course description, if not the course title, includes the term "database" or named specific types of databases, including SQL (or MySQL, NoSQL, SQLite, etc.) or graph databases.

1: No course covering material.

- Mathematical Representations

4: Required course covering linear algebra or representation theory.

2: Optional course covering linear algebra or representation theory.

1: No course covering material.

3. Computing with Data

4: Required course(s) covering multiple computer languages and/or interfaces, such as high performance computing or cloud computing.

3: Required or optional course covering at least one computer language and/or interfaces, such as high performance computing or cloud computing, and optional course mentioning coverage of an additional language.

2: Optional course(s) covering one or more computer languages and/or interfaces, such as high performance computing or cloud computing.

1: No course covering material.

4. Data Modeling

- Generative modeling

4: Required course focuses on machine/statistical learning or subarea of machine/ statistical learning.

3: Required course description includes some machine/statistical learning topics, such as decision trees, clustering, classification, etc.; however, machine/statistical learning was not the primary focus of the course.

2: Optional course focuses on machine/statistical learning or subarea of machine/ statistical learning or includes some machine/statistical learning topics, such as decision trees, clustering, classification, etc.

1: No course covering material.

- Predictive modeling

4: Required course in machine/statistical learning or subarea of machine/statistical

learning; or required course covering logistic regression.

3: Required course includes some machine/statistical learning topics, such as decision trees, clustering, classification; however, machine/statistical learning was not the primary focus of the course.

2: Optional course in machine/statistical learning; or optional course includes some

machine/statistical learning topics, such as decision trees, clustering, classification; however, machine/statistical learning was not the primary focus of the course; or optional course covering logistic regression.

1: No course covering material.

5. Data Visualization and Presentation

4: Required course covering data visualization or information visualization in data- intensive courses.

3: Required course covering visualization of information, but not data visualization.

2: Optional course covering data visualization or information visualization in

data-intensive courses.

1: No course covering material.

6. Science about Data Science

4: Required course covering evaluation of trends in, assessment of, and comparison among data science applications.

3: Required course covering data science best practices.

2: Required or optional course provides broad overview of data science methods; or

optional course covering evaluation of trends in data science applications or data science

best practices.

1: No course covering material.

**NA Framework, after National Academies of Sciences, Engineering, and Medicine (2018)**

1. Mathematical foundations

- Set theory and basic logic

4: Required course description includes explicit term "set theory" or the term "set," with a determination that this course is covering set theory.

2: Optional course includes explicit term "set theory" or the term "set," with a determination that this course is covering set theory.

1: No course covering material.

- Multivariate thinking via functions and graphical displays

4: Required course covering multivariable calculus, multivariate calculus, calculus with multiple variables, vector calculus, or multivariable/multivariate functions.

2: Optional course covering multivariable calculus, multivariate calculus, calculus with multiple variables, vector calculus, or multivariable/multivariate functions.

1: No course covering material.

- Basic probability theory and randomness

4: Required course covering probability theory or probability.

3: Required introductory statistics course.

2: Optional course covering probability theory, probability, or introductory statistics.

1: No course covering material.

- Matrices and basic linear algebra

4: Required course covering linear algebra.

2: Optional course covering linear algebra.

1: No course covering material.

- Networks and graph theory

4: Required course description includes terms "graph theory," "theory of graphs," or closely related terms. The term "graph" or "graphs" when used alone receives full credit if it is judged that the course would cover graph theory, such as in a course on discrete mathematics or discrete structures. The mention of "network" receives full credit in a required course description when it is judged that there is sufficient emphasis on the mathematical foundations of graphs.

2: Optional course description includes terms "graph theory," "theory of graphs," or closely related terms. The term "graph" or "graphs" when used alone receives full credit if it is judged that the course would cover graph theory, such as in a course on discrete mathematics or discrete structures. The mention of "network" receives full credit in a required course description when it is judged that there is sufficient emphasis on the mathematical foundations of graphs.

1: No course covering material.

- Optimization

4: Required course covering optimization.

2: Optional course covering optimization.

1: No course covering material.

2. Computational foundations

- Basic abstractions

4: Required computer science course.

2: Optional computer science course.

1: No course covering material.

- Algorithmic thinking

4: Required course with computer programing application covering algorithmic thinking. Terminology receiving full credit includes "computational thinking," "developing algorithms," and "design and problem solving."

2: Optional course with computer programming application covering algorithmic thinking. Terminology receiving equivalent credit includes "computational thinking," "developing algorithms," and "design and problem solving."

1: No course covering material.

- Programming concepts

4: Required course in computer science, covering programming language (i.e. Python, R, C++, Java, etc.) and not an interface (i.e., Excel, JMP, etc.).

3: Required course having students use a programming language (such as a bioinformatics course using Python) but not covering the programming language explicitly.

2: Optional course either explicitly covering or having students use a programming language.

1: No course covering material.

- Data structures

4: Required course in computer science or computer programming.

3: Required applications courses in computer science or computer programming that did not explicitly teach data structures.

2: Optional course in computer science or computer programming, or optional applications courses in computer science or computer programming that did not explicitly teach data structures.

1: No course covering material.

- Simulations

4: Required course covering simulations.

2: Optional course covering simulations

1: No course covering material.

3. Statistical foundations

- Variability, uncertainty, sampling error, and inference

4: Required statistics course.

2: Optional statistics course.

1: No course covering material.

- Multivariate thinking

4: Required statistics course description includes term "multivariate" or another term indicating coverage of multivariate thinking, such as discriminant analysis or principal components analysis.

2: Optional course description includes term "multivariate" or another term indicating coverage of multivariate thinking, such as discriminant analysis or principal components analysis.

1: No course covering material.

- Nonsampling error, design, experiments (e.g., A/B testing), biases, confounding, and causal inference

4: Required statistics course description includes terms "experimental design," "design," or something similar.

2: Optional statistics course description includes terms "experimental design," "design," or something similar.

1: No course covering material.

- Exploratory data analysis

4: Required statistics course, or explicit mention of "exploratory data analysis" in required course description.

2: Optional statistics course, or explicit mention of "exploratory data analysis" in optional course description.

1: No course covering material.

- Statistical modeling and model assessment

4: Required statistics course description includes term "model."

3: Required statistics course.

2: Optional statistics course.

1: No course covering material.

- Simulations and experiments

4: Required course covering both simulations and experiments.

3: Required course covering simulations or experiments.

2: Optional course covering simulations and/or experiments.

1: No course covering material.

4. Data management and curation

- Data provenance

4: Required course description specifically mentioning "provenance" or some terminology encompassing history of data/data sources.

2: Optional course description specifically mentioning "provenance" or some terminology encompassing history of data/data sources.

1: No course covering material.

- Data preparation, especially data cleansing and data transformation

4: Required course covering data preparation, especially data cleansing, data transformation, or similar language.

2: Optional course covering data preparation, especially data cleansing, data transformation, or similar language; or required course description specifically mentioning"data ingestion."

1: No course covering material.

- Data management (of a variety of data types)

4: Required course covering data management.

2: Optional course covering data management.

1: No course covering material.

- Record retention policies

4: Required course covering data storage, archives, or consideration of the ethics of data. The focus is on acknowledging the ethics or sensitivity of data.

2: Optional course covering data storage, archives, or consideration of the ethics of data. The focus is on acknowledging the ethics or sensitivity of data; however, ethics or sensitivity of data is not the primary focus of the course.

1: No course covering material.

- Data subject privacy

4: Required course covering privacy, ethics of data, etc.

2: Optional course covering privacy, ethics of data, etc.

1: No course covering material.

- Missing and conflicting data

4: Required course covering missing data.

2: Optional course covering missing data.

1: No course covering material.

- Modern databases

4: Required course description, if not the title, includes the term "database" or specific types of databases, such as SQL (or MySQL, NoSQL, SQLite, etc.) or graph databases.

2: Optional course description, if not the title, includes the term "database" or specific types of databases, such as SQL (or MySQL, NoSQL, SQLite, etc.) or graph databases.

1: No course covering material.

5. Data description and visualization

- Data consistency checking

4: Required statistics course.

2: Optional statistics course.

1: No course covering material.

- Exploratory data analysis

4: Required statistics course, or explicit mention of "exploratory data analysis" in required course description.

2: Optional statistics course or explicit mention of "exploratory data analysis" in optional course description.

1: No course covering material.

- Grammar of graphics

4: Required course covering grammar of graphics.

3: Required course in data visualization or, for data-intensive courses, information visualization.

2: Optional course covering grammar of graphics or data visualization; or, for data-intensive courses, information visualization.

1: No course covering material.

6. Data modeling and assessment

- Machine learning

4: Required course in machine learning or subarea of machine learning.

2: Optional course in machine learning or subarea of machine learning.

1: No course covering material.

- Multivariate modeling and supervised learning

4: Required course in machine learning, statistical learning, or supervised learning; or

required course description including common machine learning terms, such as decision trees and clustering.

3: Required course in multivariate modeling.

2: Optional course in machine learning, statistical learning, or multivariate modeling; or optional course description including common machine learning terms, such as decision trees and clustering; or optional course in multivariate modeling.

1: No course covering material.

- Dimension reduction techniques and unsupervised learning

4: Required course(s) covering both dimension reduction techniques and unsupervised learning, not necessarily in the same course. Other terms counting for coverage of dimension reduction techniques includes principal components analysis and discriminant analysis.

3: Required course covering either dimension reduction techniques or unsupervised learning. Other terms counting for coverage of dimension reduction techniques includes principal components analysis and discriminant analysis.

2: Optional course covering either dimension reduction techniques or unsupervised

learning. Other terms counting for coverage of dimension reduction techniques includes principal components analysis and discriminant analysis.

1: No course covering material.

- Deep learning

4: Required course has as a focus deep learning, neural networks, or something very similar (e.g. deep architectures).

3: Required course covering to some extent (as a minor topic) deep learning, neural networks, or something very similar (e.g., deep architectures).

2: Optional course covering at least to some extent (as a minor topic) deep learning, neural networks, or something very similar (e.g., deep architectures)

1: No course covering material.

- Model assessment and sensitivity analysis

4: Required course description specifically mentioning "model assessment," "model selection," "model comparison," "model evaluation," or something very similar whereby models were compared to one another or judged for quality.

3: Required course covering statistical modeling.

2: Required statistics course, without additional explanation; or optional course description specifically mentioning "model assessment," "model selection," "model building," "model comparison," "model evaluation," or something very similar; or optional course covering statistical modeling.

1: No course covering material.

- Model interpretation (particularly for black box models)

4: Required machine/statistical learning (or subarea of machine/statistical learning) course; or required course description including common machine/statistical learning terms, such as decision trees and clustering.

2: Optional machine/statistical learning (or subarea of machine/statistical learning)

course; or optional course description including common machine/statistical learning terms, such as decision trees and clustering.

1: No course covering material.

7. Workflow and reproducibility

- Workflows and workflow systems

4: Required course description in statistics or data science specifically mentioning "workflows," "pipelines," or similar term(s).

2: Optional course description in a domain outside statistics or data science (e.g., computer science) specifically mentioning "workflows," "pipelines," or similar term(s).

1: No course covering material.

- Reproducible analysis

4: Required course description in statistics or data science specifically mentioning reproducibility, repository, documenting work, etc.

2: Required course description in a domain outside statistics or data science (e.g., computer science) specifically mentioning "workflows," "pipelines," or similar terms; or optional course description in statistics or data science specifically mentioning reproducibility, repository, documenting work, etc.

1: No course covering material.

8. Communication and teamwork

- Ability to understand client needs

4: Required course description mentioning interaction with clients.

2: Required internship; or optional course mentioning interaction with clients.

1: No course covering material.

- Clear and comprehensive reporting

4: Required course description specifically mentioning teaching of reporting skills.

3: Required course has report, application paper, application presentation, or emphasis on communication as component, but there is no discussion of teaching reporting skills.

2: Default score.

- Conflict resolution skills

4: Required course description specifically mentioning teaching of conflict resolution

skills, etc.

3: Required course has team project as component, but does not specifically mention teaching conflict resolution skills.

2: Default score.

- Well-structured technical writing without jargon

4: Required course covering technical writing.

3: Required course includes a significant writing component; or optional course provides instruction on technical writing.

2: Default score.

- Effective presentation skills

4: Required course on communication or teaching presentation skills.

3: Required course includes presentation as course component, but no mention of teaching presentation skills; or optional course on communication or teaching presentation skills.

2: Default score.

9. Domain-specific considerations

4: Required major in domain outside of computer science, mathematics, or statistics.

3: Required minor or five domain-specific courses to effectively "equal" a minor outside

of computer science, mathematics, or statistics.

2: Required additional courses for major that are not computer science, mathematics, or statistics courses and go beyond a program’s general education requirements.

1: No additional requirements, or only required general education courses; or absence of required courses that are not computer science, mathematics, or statistics courses for major.

10. Ethical Problem Solving

- Ethical precepts for data science and codes of conduct

4: Required course(s) covering ethical precepts for data science and codes of conduct.

3: Required course(s) introducing ethical precepts for data science and codes of conduct, but not at the level necessary to receive a 4.

2: Optional course covering ethical precepts for data science and codes of conduct.

1: No course covering material.

- Privacy and confidentiality

4: Required course(s) covering privacy, confidentiality, or ethics of data.

3: Required course(s) introduced privacy, confidentiality, or ethics of data, but not at the level necessary to receive a 4.

2: Optional course covering privacy, confidentiality, or ethics of data.

1: No course covering material.

- Responsible conduct of research

4: Required course description specifically mentioning responsible conduct of research or something similar.

3: Required course description mentioning responsible conduct of research or something similar, but not at the level necessary to receive a 4.

2: Optional course description specifically mentioning responsible conduct of research or something similar.

1: No course covering material.

- Ability to identify "junk" science

4: Required course description specifically mentioning junk science or pseudoscience.

3: Required course description including language like "critically evaluating the quality of prior published work."

2: Required course description explicitly mentioning the scientific method; or optional course description including language like "critically evaluating the quality of prior published work."

1: No course covering material.

- Ability to detect algorithmic bias

4: Required course description explicitly mentioning algorithmic bias, ethics of algorithm development/application, or something of that ilk.

2: Optional course description explicitly mentioning algorithmic bias, ethics of algorithm development/application, or something of that ilk.

1: No course covering material.
